# A comprehensive catalog of predicted functional upstream open reading frames in humans

**DOI:** 10.1093/nar/gky188

**Published:** 2018-03-19

**Authors:** Patrick McGillivray, Russell Ault, Mayur Pawashe, Robert Kitchen, Suganthi Balasubramanian, Mark Gerstein

**Affiliations:** 1 Molecular Biophysics and Biochemistry Department, Yale University, New Haven, CT 06520, USA; 2 Program in Computational Biology and Bioinformatics, Yale University, New Haven, CT 06520, USA; 3 Department of Computer Science, Yale University, New Haven, CT 06520 USA

## Abstract

Upstream open reading frames (uORFs) latent in mRNA transcripts are thought to modify translation of coding sequences by altering ribosome activity. Not all uORFs are thought to be active in such a process. To estimate the impact of uORFs on the regulation of translation in humans, we first circumscribed the universe of all possible uORFs based on coding gene sequence motifs and identified 1.3 million unique uORFs. To determine which of these are likely to be biologically relevant, we built a simple Bayesian classifier using 89 attributes of uORFs labeled as active in ribosome profiling experiments. This allowed us to extrapolate to a comprehensive catalog of likely functional uORFs. We validated our predictions using in vivo protein levels and ribosome occupancy from 46 individuals. This is a substantially larger catalog of functional uORFs than has previously been reported. Our ranked list of likely active uORFs allows researchers to test their hypotheses regarding the role of uORFs in health and disease. We demonstrate several examples of biological interest through the application of our catalog to somatic mutations in cancer and disease-associated germline variants in humans.

## INTRODUCTION

**Figure 1.**
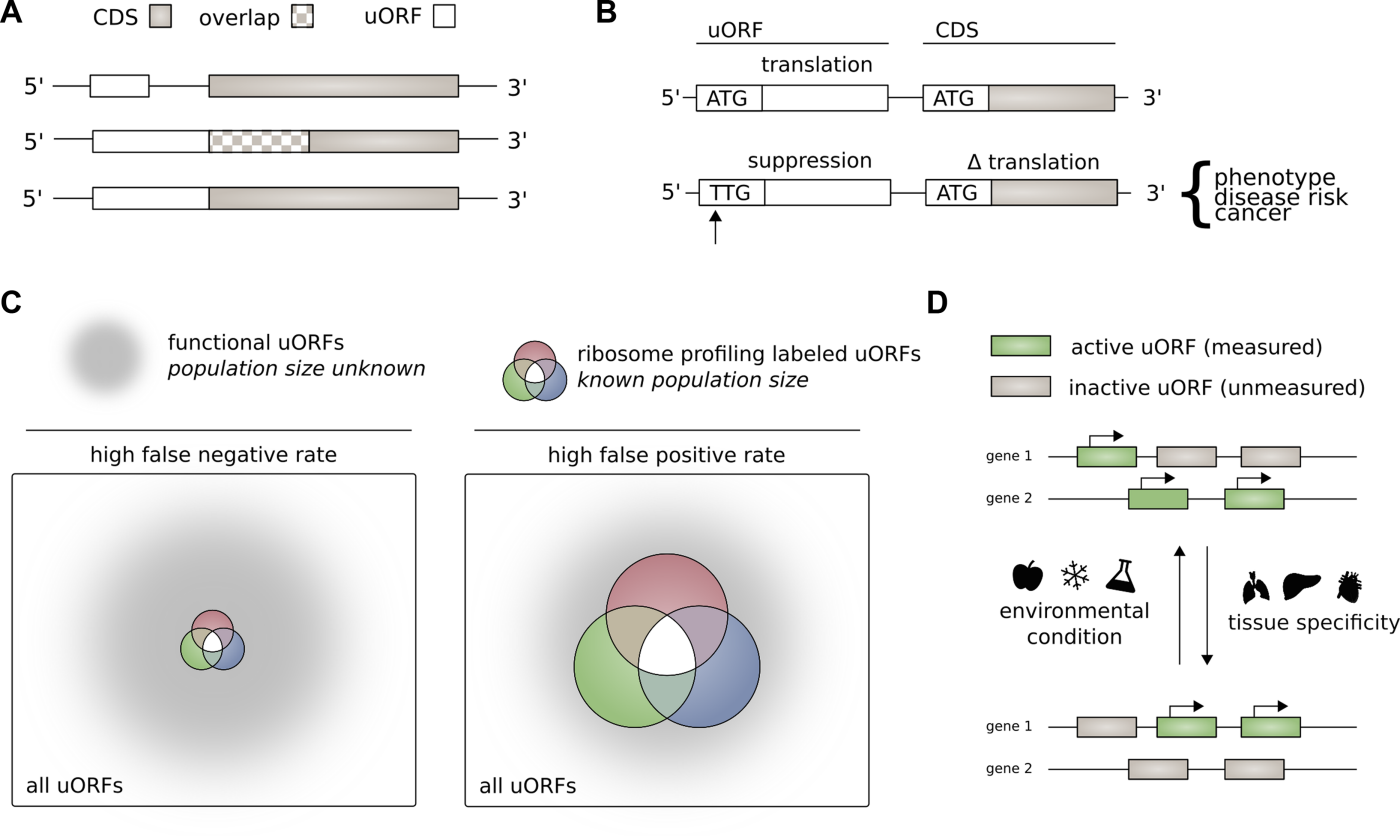
(**A**) Structure of upstream open reading frames. The stop codon of an uORF may be located before the CDS start codon [top], or downstream of the CDS start codon if the uORF is frame-shifted relative to the CDS [middle]. If the uORF and CDS share the same stop codon, the uORF acts as a 5′ extension of the CDS [bottom]. (**B**) Effect of mutation or variation on upstream open reading frames. Creation or destruction of an upstream open reading may have a downstream effect on translation of the coding sequence. Change in the translation of the coding sequence may result in a change in phenotype and disease risk. (**C**) Sensitivity and specificity of ribosome profiling for identifying upstream open reading frames. It is possible that ribosome profiling studies have a high false negative rate (left), or a high false positive rate (right). We make the assumption that ribosome profiling studies have a high false negative rate for identifying translated upstream open reading frames (left). (**D**) The activity of uORFs varies according to cell type and environmental stimuli. uORFs may not be detected in a ribosome profiling experiment due to variation in uORF activity with cell type and cell environment.

Upstream open reading frames (uORFs) consist of a start codon in the 5′ untranslated region of a gene (UTR) and an associated stop codon appearing before the stop codon of the main coding DNA sequence (CDS). An uORF may begin and end before the main gene coding sequence. Alternatively, if the upstream reading frame is out of frame with the CDS, it may overlap with the CDS (Figure 1A). uORFs are latent in mRNA transcripts and may undergo translation.

An initial survey of the human genome identified uORFs contained in ∼10% of mRNA transcripts (1). More recent analyses identify uORFs in association with nearly half of all mRNA transcripts (2). The discovery that many translated uORFs utilize near-cognate start codons to the canonical ATG start codon has broadened estimates of uORF prevalence further (3–7).

Presence of functional uORFs is generally thought to suppress translation of downstream genes (8–13) (Figure 1B). Proposed molecular mechanisms for modification of CDS translation by uORFs are numerous. These include *translation reinitiation –* the uORF and CDS are translated by the same ribosome in series—*leaky-scanning*—ribosome recognition of an uORF and subsequent CDS translation, without uORF translation—and *ribosome-stalling*—decreased translation of the CDS due to ribosome retention at the upstream uORF (3,7,14). Differential translation of multiple protein products may occur in consequence to an uORF (15). It is also possible for an uORF to function as a short open reading frame, encoding a functional peptide (16–19). uORF function is not necessarily constant—uORFs may display differential function in stressed cells compared with non-stressed controls (20–25).

Study of uORF translation and function was historically limited to the experimental evaluation of individual uORFs (8,26). Genome-scale ribosome profiling studies have allowed for the identification of large populations of uORFs known to undergo translation (4,27,28). This mapping of translation initiation is sufficient to make an association between ribosomes and particular start codons and reading frames (29–31).

We proceed on the assumption that the total universe of active uORFs is much larger than that identified through ribosome profiling experiments. In other words, we assume that ribosome profiling experiments have high specificity in identifying functional uORFs with a high false-negative rate (Figure 1C). Ribosome profiling experiments follow a challenging technical procedure, and it is uncertain whether all potentially active uORFs are measurable in a given sample (Figure 1D). This is consistent with a high false-negative rate. Other researchers have implicitly endorsed this hidden assumption when predicting translated uORFs in *Saccharomyces cerevisiae* and *Arabidopsis thaliana*, on the basis of DNA sequence and ribosome profiling data (32,33). A similar assumption is the basis for using patterns of ribosome profiling occupancy to maximize the number of inferred translation products in humans (34,35). Although many uORFs may indeed be functionally neutral – neither translated nor affecting the activity of the downstream CDS – we sought to distinguish active uORFs from functionally neutral uORFs on the basis of specific attributes.

For our investigation of the prevalence of active uORFs in humans, we began with a genome-wide scan, searching for uORFs associated with protein-coding genes listed in the GENCODE genome annotation (36). uORFs beginning with ATG or a single nucleotide variant of ATG were identified. This scan yields a universe of uORFs numbering nearly 1.3 million.

uORFs in this large set were classified as active according to similarity to uORFs occupied in ribosome profiling experiments. This classification was accomplished using a Naïve-Bayes classifier, trained on 89 uORF attributes. We validated our predicted uORFs using a cross-validation method where two ribosome profiling experiments are used to predict the uORFs translated in a third experiment. We also validated our predictions by examining how gene level protein expression and local ribosome activity correlate with genetic variants that alter uORFs in 46 individuals.

The 1000 Genomes Project's database of human variation (37) and the NHGRI-EBI GWAS catalog (38) were used to provide a baseline for the functional consequence of our predicted active uORFs. The predictions we generated were also used to measure the functional impact of somatic mutations affecting uORFs in tissue-matched tumor samples (39).

We provide a resource of predicted active uORFs for other scientists to use in their effort to understand uORF function in health and disease.

## MATERIALS AND METHODS

### Extracting uORFs from GENCODE

uORFs were identified through genome-wide search performed on GENCODE Release 19, GENCODE’s GRCh37 human genome annotation (36). uORFs were defined as a start codon within the 5′UTR and a downstream stop codon before the end of the CDS. All three possible reading frames were examined. ATG and near-cognate start codons were included in this search [ATG, TTG, GTG, CTG, AAG, AGG, ACG, ATA, ATT, ATC]. The decision to scan all near-cognate start codons was motivated by prior investigations that highlight significant uORF translation initiation at near-cognate start codons (3–7).

### Ribosome profiling experiments as a reference set

Three independent ribosome profiling experiments performed by Lee *et al.* (31), Fritsch *et al.* (30), and Gao *et al.* (29) were used to obtain an experimentally validated set of translated upstream open reading frames. All three of these studies identified translation initiation sites (TIS) through treatment of human cell lines with antibiotic translation inhibitors. These antibiotic treatments are designed to halt ribosomes in proximity to the start codon (12–13 nucleotides downstream). As such, these experiments can provide high-resolution information about translation initiation sites in the human genome. The studies of Lee *et al.* and Gao *et al.* were performed on the HEK293 human cell line. The study of Fritsch *et al.* was performed on the THP-1 human cell line. All three experiments identified uORFs and included non-cognate start codons. These three experiments thus provide a platform for identifying common features of translated uORFs between experiments in the same tissue and also across tissue-types, under a shared annotation framework.

### A literature review of translated human uORFs

In addition to ribosome profiling studies, confirmed translated uORFs were obtained from the biomedical literature (8,40,41). uORFs studied in humans that displayed functionality – demonstrated regulation of the CDS product – were added to the set of positive uORFs. In total, 33 uORFs associated with 33 separate genes were included from this literature review.

### Cleansing the dataset, by removal of N-terminal extensions and alternative translation initiation sites (aTISs), and isolation of unique uORFs

N-terminal extensions of the CDS sequence may retain some functional activity of the primary gene protein product and were removed from the dataset. Any uORF start codon annotated as an alternative translation initiation site (aTIS) for the CDS was also removed from the dataset.

A single uORF may be present on multiple transcripts. In order to avoid over-counting, uORFs were distinguished on the basis of their unique genomic coordinates. When a single uORF was present on multiple transcripts, we associated one transcript with the coordinates of that uORF as an identifier.

### 1-voted, 2-voted and unlabeled datasets

uORFs were divided into three separate sets according to their experimental translation status:

*2-voted:* uORFs identified as translated in two or more ribosome profiling experiments, or through literature review.
*1-voted:* uORFs identified as translated in only one ribosome profiling experiment, or through literature review.
*Unlabeled:* uORFs that were not identified as translated in any ribosome profiling experiment, or through literature review.

### Ribosome binding strength of uORF start codons

We obtained transcript-level expression data from The Human Protein Atlas for the cell lines THP-1 and HEK 293 and used this data to normalize the footprinting reads from each experiment (42).

According to the metric of normalized footprinting reads per start codon, we classified uORFs as (a) strong ribosome binding strength uORFs (>50th percentile binding strength among positive uORFs) and (b) weak ribosome binding strength uORFs (<50th percentile among positive uORFs). We then compared the experimental translation status for high binding strength uORFs to low binding strength uORFs.

### Mass spectrometry (MS) evidence of uORF translation

We obtained short peptide sequences from The PeptideAtlas Project Tiered Human Integrated Search Proteome (THISP) database (43). This database pulls major sources of human protein sequences identified in MS experiments into an integrated resource. We used the 1 October 2016 build of the Human PeptideAtlas which contains 1,222,862 unique peptide sequences.

We then performed a BLAST (blastx) search (44) of this peptide database against our genome-wide scan of uORFs from the GENCODE annotation. We required that positive matches have an expect (*E*) value <1 × 10^−5^, with no gaps in the mapped sequence, and occur in the same reading frame as the uORF. We also required that our positive matches not map to CDS regions.

### Estimating the total population of active uORFs

Based on observed overlap among ribosome profiling experiments, an estimate for the total number of active uORFs was made using methods from population biology. In general, upon marking a number of items (*M*) in a population of size *N*, the number of marked items (*R*) in a randomly drawn subsequent sample of size *C* will roughly reflect the proportion of marked items in the total population, i.e.:

(1) \begin{equation*}\frac{R}{C} \cong \frac{M}{N}\end{equation*}

A simple rearrangement yields an estimate of the total population size known as the Petersen estimate (45):

(2) \begin{equation*}{\boldsymbol{\hat{N}}} = \frac{{CM}}{R}\end{equation*}

where $\hat{N}$ is the population size estimate. We treated functional uORFs identified in ribosome profiling experiments as samplings of the total population of functional uORFs. If ribosome profiling experiments are examined sequentially such that functional uORFs identified in one experiment may be reidentified in subsequently examined experiments, the Schnabel equation (Equation 3) or Schumacher and Eschmeyer equation (Equation 4) provide a means of combining the multiple samplings to estimate the total functional uORF population size (46,47). The Schnabel equation is a weighted average of Petersen estimates:

(3) \begin{equation*}{\boldsymbol{\hat{N}}} = \frac{{\mathop \sum \nolimits_{{\boldsymbol{t}} = 1}^{\boldsymbol{S}} ({{\boldsymbol{C}}_{\boldsymbol{t}}}{{\boldsymbol{M}}_{\boldsymbol{t}}})}}{{\mathop \sum \nolimits_{{\boldsymbol{t}} = 1}^{\boldsymbol{S}} {{\boldsymbol{R}}_{\boldsymbol{t}}}}}\end{equation*}

where *S* is a series of ribosome profiling experiments with t $ \in$ {1…*S*}, ${C_t}$ the number of functional uORFs in a sample, ${M_t}$ the cumulative number of functional uORFs identified prior to sampling *t*, and ${R_t}$ the number of functional uORFs reidentified in sample *t*. Similarly, the Schumacher and Eschmeyer equation takes multiple Petersen estimates to provide a series of data points, ${M_t}$ (x-coordinates) and ${R_t}/{C_t}$ (y-coordinates). A least-squares fit to these data points is an estimate of the inverse of the population size:

(4) \begin{equation*}{\boldsymbol{\hat{N}}} = \frac{{\mathop \sum \nolimits_{{\boldsymbol{t}} = 1}^{\boldsymbol{S}} ({{\boldsymbol{C}}_{\boldsymbol{t}}}{\boldsymbol{M}}_{\boldsymbol{t}}^2)}}{{\mathop \sum \nolimits_{{\boldsymbol{t}} = 1}^{\boldsymbol{S}} {{\boldsymbol{R}}_{\boldsymbol{t}}}{{\boldsymbol{M}}_{\boldsymbol{t}}}}}\end{equation*}

### Extraction of attributes associated with uORFs

Feature data were extracted for each uORF. Features were chosen to cover a broad range of categories of data, including features associated with uORF position and length, conservation, functional metrics like RNA expression, and sequence-based signatures that may relate to translation. Eighty nine features were used in total. A complete listing of these features including descriptions is included as Supplemental Table S1.

### Feature discretization

The minimum description length principle (MDLP) algorithm was used to discretize each of our chosen attributes (48). The MDLP algorithm minimizes information lost through discretization. MDLP discretization was implemented using the ‘discretization’ package available for R (http://cran.r-project.org/web/packages/discretization/index.html).

### Prioritization of feature data

The distribution for each feature was compared between positive and unlabeled uORFs using the Kolmogorov–Smirnov (KS) statistic. A greater KS statistic suggests the greater ability of that attribute to distinguish between positive and unlabeled uORFs.

### Classifying uORFs according to attributes

We determined that attributes of an uORF were consistent with an active uORF according to a Naïve-Bayes machine learning algorithm applied to positive and unlabeled examples (49):

(5) \begin{equation*}{{P}_{{pos}}}{\ }\mathop \prod \limits_{{i\ } = {\ }1}^{N} {p}\left( {{{A}_{i}}{\rm{|}}{pos}} \right) = {{p}_{{pos}}}\end{equation*}

(6) \begin{equation*}{P_{neg}}\mathop \prod \limits_{i\ = \ 1}^N p\ \left( {{A_i}{\rm{|}}unl} \right) = {p_{neg}}\ \end{equation*}

where:

(7) \begin{equation*}{P_{neg}} + \ {P_{pos}} = \ 1\end{equation*}

${P_{pos}}$ is the prior probability associated with positive uORFs. ${P_{pos}}$ was chosen as the F1 score maximizing value (0.61). $p( {{A_i}{\rm{|}}pos} )$, and $p( {{A_i}{\rm{|}}unl} )$ represent the frequency of that attribute value among the positive and unlabeled sets respectively. ${p_{pos}}$ represents the probability the uORF is positive. ${p_{neg}}$ represents the probability the uORF is negative. We labeled an uORF as positive or negative according to the greater value between ${p_{pos}}$ and ${p_{neg}}$. Consistent with this labeling, we scored the uORFs as ${\rm log}( {\frac{{{p_{pos}}}}{{{p_{neg}}}}} )$ with a score of 0 as the threshold between positive and negative uORFs. We note likely violation of the feature independence requirement of Naïve-Bayes. However, empirical and theoretical study has demonstrated optimal classification performance, even where feature independence does not hold (50,51).

### Peptide feature score

In addition to our uORF functional classification, we also calculated a peptide score for each uORF based on protein features that may be relevant to translated uORF peptides. The included features were protein length, the 20 amino acid frequencies, and evidence of protein translation from MS (Supplemental Table S1). These features were combined using a Naïve-Bayes methodology similar to our main uORF score.

### Translation at lncRNA ORFs

In order to begin an investigation of the function of translation of ORFs on lncRNA, we first completed genome-wide identification of ORFs on lncRNA using the GENCODE Release 19 lncRNA annotation.

The studies of Fritsch *et al.*, Lee *et al.* and Gao *et al.* do not comment on lncRNA translation initiation sites. We identified ORF translation sites on lncRNAs from these three studies as follows: after downloading signal tracks for these profiling experiments from the GWIPsViz browser (52), we intersected our genome-wide lncRNA ORFs with ribosome profiling reads. Similar to the threshold used by Lee *et al.*, we identified the ORF as translated if ≥10 reads mapped to the start codon within ±1 nucleotide. Similar to our analyses of uORFs on coding transcripts, we identified a gold-standard positive set of ORFs as lncRNA ORFs that are found translated in at least two of three experiments.

### Model validation

Our model was serially trained on two of three ribosome profiling datasets, using the trained model to extract the third withheld ribosome profiling dataset from among the unlabeled examples. The success of differentially trained models in this cross-validation was evaluated using ROC curves, with an area under the curve (AUC) calculated for each curve.

As a biologic validation of our predicted uORFs, we examined the effect of alteration of a predicted active uORF’s start codon on gene protein levels and local ribosome occupancy. Both protein quantitation and local ribosome quantitative trait loci (*cis*-rQTL) for 62 Yoruba lymphoblastoid cell lines are available from the ribosome profiling and proteomic experiments of Battle *et al.* (53). SNV array data is available for all 62 individuals (54). However, in order to evaluate the greatest possible range of polymorphisms we chose to only examine 46 of the 62 individuals for whom variant calling based on whole genome sequencing is available through the 1000 Genomes Project. Protein expression change was evaluated in association with both gain of predicted positive uORFs (ATG and CTG) and loss of predicted positive uORFs. We analyzed uORF start codon gain and loss because of the clear relationship to uORF gain and loss. This is different from uORF stop codon gain and loss, which generally results in a change in length of the uORF sequence (uORF truncation and elongation). A change in length of a uORF may affect the translation of downstream coding sequences (e.g. via change in rate of leaky scanning or translation reinitiation). However, the effect of extension or truncation of a uORF on translation of the main CDS depends greatly on local context and is challenging to interpret in aggregate. Functional annotation clustering of genes associated with variants examined was performed using Database for Annotation, Visualization and Integrated Discovery v.6.8 (DAVID) (55).

### Natural variation and somatic mutation affecting predicted positive uORFs

Single nucleotide variants (SNVs) that affect the start codons of predicted positive uORFs were identified using data from the 1000 Genomes Project Phase 3 callset (56). Measurement of comparative frequency of mutation among uORF start codons was taken as a measure of evolutionary conservation and functional significance of predicted positive uORFs. Ultra-rare, singleton variants from the Exome Aggregation Consortium (ExAC) catalog v.1 were also examined for their effect on predicted positive uORFs (57). A subset of SNVs affecting uORFs that are associated with disease and differential disease susceptibility were identified through a search of the NHGRI-EBI GWAS database (58), the Human Gene Mutation Database (HGMD) of published human inherited disease mutations (59), and the ClinVar database of variants with human phenotypic correlations (60).

Furthermore, the study of Alexandrov *et al.* (39) provides a set of exomic somatic mutations by patient sample and cancer type. We used these mutations as a comparison standard for the healthy 1000 Genomes Project population. We identified start codons of our predicted positive uORFs altered by somatic mutation in cancer. These somatic mutations were annotated and prioritized according to their recurrence in patient samples, and according to their effect on Catalogue of Somatic Mutations in Cancer (COSMIC) cancer genes (61).

## RESULTS

*In silico* identification based on a genome-wide search using the GENCODE Release 19 gene annotation model yielded 1,270,265 unique uORFs. Within this large set, we extracted the subset of uORFs identified as translated in the studies of Lee *et al.*, Fritsch *et al.* and Gao *et al.* We further stratified this set of translated uORFs according to shared representation of uORFs among the three studies. uORFs identified in the intersection between two or more of these studies were used as the reference standard for functional uORFs. Literature review yielded 33 additional examples of active uORFs that were also included in the set of positive, functional uORFs.

**Figure 2.**
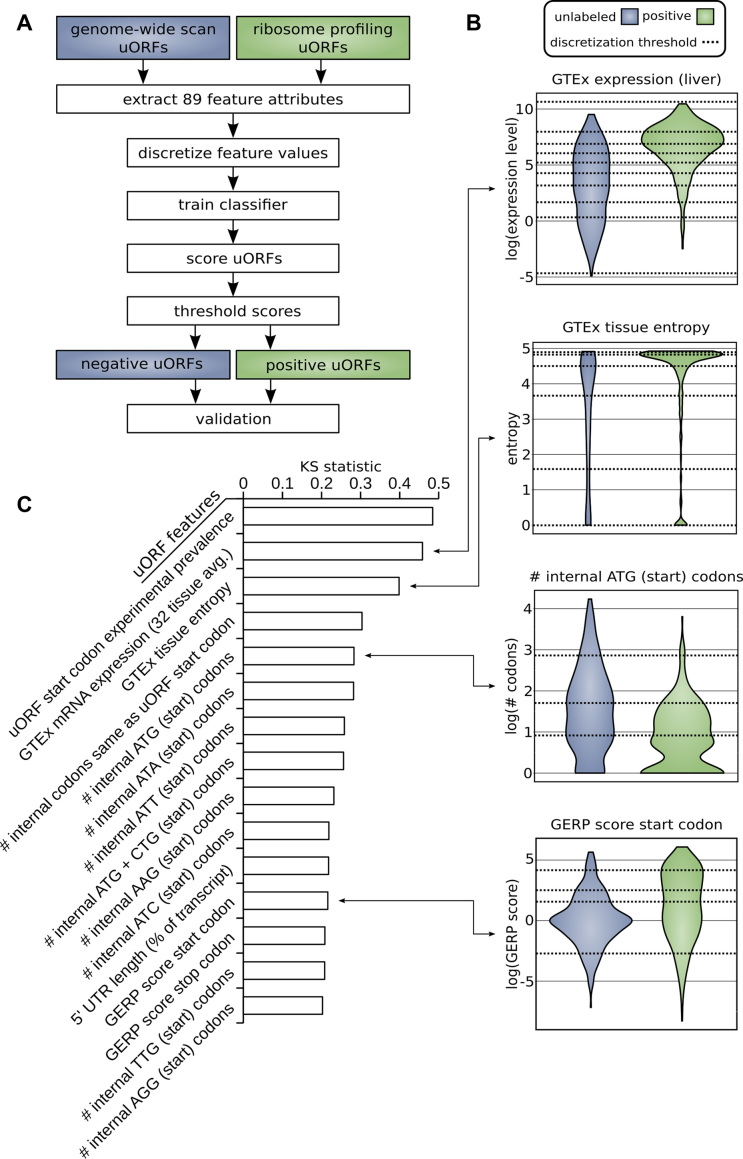
(**A**) Methodology for distinguishing positive from unlabeled uORFs. uORFs identified through genome-wide scan and uORFs labeled in ribosome profiling experiments were used to train a machine learning algorithm to identify uORFs that are likely active (positive predictions). (**B**) Examples of differential distributions of attributes between positive and unlabeled uORFs. uORF attributes are used to distinguish positive from unlabeled uORFs. Continuous distributions were discretized and optimized for machine learning using the minimum description length principle (MDLP) binning algorithm. Horizontal lines on the plot correspond to these binning intervals. (**C**) Upstream open reading frame attribute ranking. Attributes are ranked according to the difference in distribution between positive and unlabeled uORFs using the KS statistic. The top 15 features according to this prioritization are shown.

We followed the procedure outlined in Figure 2A to identify uORFs that are likely to be active. Distributions of attributes for positive, translated uORFs were compared with distributions of those same attributes observed in the set of unlabeled uORFs (Figure 2B). The KS statistic and corresponding *P*-value for each of the 89 attributes assessed in this study are provided in Supplemental Table S2. The top 15 attributes listed according to the magnitude of KS statistic are given in Figure 2C. This prioritization of features suggests how they influence the likelihood of uORF translation. Start and stop codons of functional uORFs are generally located in evolutionarily conserved sites as measured by GERP score suggesting a meaningful physiologic role. Active uORFs are on average closer to the CDS (203 nucleotides from the CDS start for positive uORFs, 318 nucleotides for unlabeled uORFs) and are associated with shorter 5′UTR regions (360 nucleotides for positive uORFs, 618 nucleotides for unlabeled uORFs). Positive uORFs also have on average fewer internal ATG and near-cognate start codons. This finding is consistent with prior work showing that longer 5′ UTRs, in general, have less functional impact on CDS translation than shorter 5′ UTRs after controlling for number of uORFs (62).

**Figure 3.**
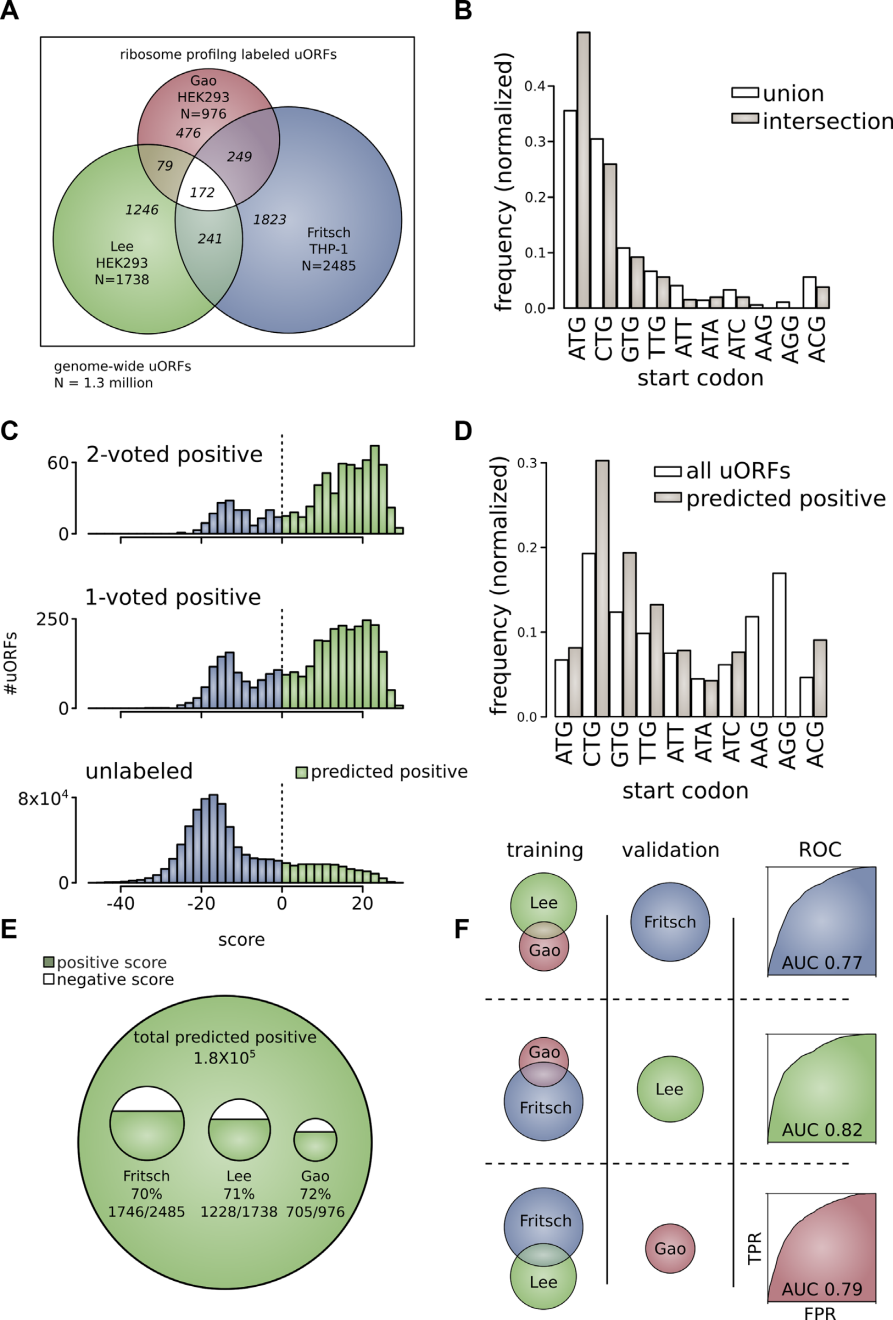
(**A**) Ribosome profiling identified uORFs as a subset of all uORFs. The universe of all uORFs is identified through comprehensive search of the GENCODE human genome annotation [outer border]. Ribosome profiling studies of Fritsch *et al.*, Lee *et al.*, and Gao *et al.* are shown as overlapping subsets of this universe. Pair-wise and three-way intersections between these experiments are highlighted. (**B**) Frequency of translated uORF ATG start codons and near-cognate start codons from ribosome profiling experiments. Frequency for uORFs translated in any experiment (union) or in more than one experiment (intersection). (**C**) Score distributions for upstream open reading frames. Score distributions for 2-voted positive uORFs that are translated in two or more ribosome profiling experiments (top), 1-voted positive uORFs that are translated in only one ribosome profiling experiment (middle), and unlabeled uORFs uncovered through genome-wide search (bottom). (**D**) The frequency of uORF ATG start codons and near-cognate start codons of predicted positive upstream open reading frames. Frequency is given for all uORFs genome-wide and for the subset of uORFs that are predicted to be active (predicted positive). (**E**) uORFs predicted as positive from genome-wide scan and ribosome profiling experiments. Approximately 180,000 uORFs in the genome are predicted as active upstream open reading frames. This large set includes substantial proportions of uORFs identified in the ribosome profiling experiments (∼70% each). (**F**) Performance of the machine learning algorithm. The machine learning algorithm was trained on two of three ribosome profiling data sets and used to extract the third data set from among unlabeled examples. The ROC curve is shown for each of the three combinations: (i) train Lee *et al.* and Gao *et al.*—extract Fritsch *et al.* (AUC = 0.77), (ii) train Fritsch *et al.* and Gao *et al.*—extract Lee *et al.* (AUC = 0.82), (iii) train Lee *et al.* and Fritsch *et al.*—extract Gao *et al.* (AUC = 0.79),

Overlap between the three ribosome profiling experiments was found to be low. 28.3% of translated uORFs from Lee *et al.* (492/1,738), 26.6% of Fritsch *et al.* (662/2,485), and 51.2% of Gao *et al.* (500/976), are positively identified in at least one other ribosome profiling experiment. The number of uORFs shared between all three sets represents only 4.0% of uORFs (172/4,286) identified in these studies (Figure 3A). In order to determine if the affinity of ribosomes for the start codons was a factor in this observed overlap, we defined a proxy of ribosome binding strength as the number of footprinting reads per start codon, normalized by transcript expression. We defined both strong binding strength uORFs (>50th percentile binding strength among positive uORFs) and weak binding uORFs and weak ribosome binding strength uORFs (<50th percentile among positive uORFs). We found there is greater percent overlap among strong binding strength uORFs compared with weak binding strength uORFs (positively identified in at least one other experiment: Lee *et al.*—19.5% weak, 39.1% strong; Fritsch *et al.*—19.5% weak, 57.4% strong, Gao *et al.*—52.3% weak, 52.5% strong).

In addition to ribosome profiling, mass spectrometry (MS) may be used to identify translated uORFs. However, prior work suggests that translated uORFs are poorly detected using MS techniques and ribosome profiling is the preferred methodology for their identification (63–65). This could be due to the fact that peptides encoded by uORFs are short and often not unique in the genome, or due to incomplete translation/rapid degradation following translation by nonsense mediated decay (NMD). With these limitations in mind, we completed a BLAST search of peptide sequences from the Human PeptideAtlas against our genome-wide uORFs extracted from the GENCODE annotation. 1,593 unique uORFs were identified with at least 1 mapped peptide sequence after removing all peptides also mapping to CDS regions. A list of uORFs with unique peptide mappings is included as Supplemental Table S3.

If independent ribosome profiling experiments represent resampling of the same population, repeat identification of uORFs among experiments yields an estimate of the total number of functional uORFs. 10,000 functional uORFs are estimated in this way to be present in the human genome using the Schnabel equation (Equation 3) or Schumacher and Eschmeyer equation (Equation 4). This estimate of the total population of functional uORFs was made with respect to uORFs defined by their unique genomic coordinates. Thus, identical uORFs across multiple transcripts are not redundantly counted.

CTG (30.5%) and ATG (34.6%) are the most prevalent start codons identified in ribosome profiling experiments. CTG (28.2%) and ATG (46.1%) continue to represent the majority of start codons in the intersection between ribosome profiling experiments (Figure 3B). Representation of every near-cognate start codon was found in intersections between studies, with the exception of AAG and AGG. This suggests that uORFs do not generally employ AAG and AGG as start codons. It has been previously suggested that identification of uORFs beginning with AAG or AGG in ribosome profiling experiments may represent false-positives (66). Discretized attributes of positive and unlabeled sets of uORFs were used to build a statistical classifier within a Naive-Bayes framework. The result of application of the classifier is shown in Figure 3C. 76.8% of 2-voted positive uORFs [590/768], 67.1% of 1-voted positive uORFs [2,379/3,543], and 14.7% of unlabeled uORFs [185,833/1,265,954] are ultimately classified as likely active. A total of 14.9% of all uORFs are identified as likely active [188,802/1,270,265]. A complete list of upstream open reading frames predicted to be active is provided as Supplemental Table S4. The 10% highest probability examples are also specified (Supplemental Table S5). In addition to our main uORF score used to predict active uORFs, we also calculated a peptide probability related to common protein features among ribosome-profiling study labelled functional uORFs (20 amino acid frequencies, peptide length, and MS evidence of translation). This peptide score was calculated separately from the main uORF score in order to distinguish post-translational features from features present in nucleotide sequences. It is presented as a probability that may be used to modify the main uORF score (see Supplemental Tables S4 and S5).

Motivated by recent ribosome profiling studies showing evidence of translation at lncRNA ORFs (3,67–69), we identified 756 unique ORFs with evidence of translation on lncRNAs, of which 174 are 2-voted uORFs with evidence of translation in two or more studies (Supplemental Table S6). The largest number of these 2-voted lncRNA ORFs exhibit translation initiation at the canonical ATG start codon (55/174). However, translation initiation was observed at all near-cognate start codons. On average, these positive (2-voted) translated lncRNA ORFs were slightly longer than uORFs—an average of 256 nucleotides for lncRNA ORFs versus 205 nucleotides for uORFs – and located slightly more distal to the 5′ cap—an average of 401 nucleotides for lncRNA ORFs versus 280 nucleotides for uORFs.

We did not apply our existing scoring algorithm to lncRNA ORFs, or include them in our training data, as there are several properties of lncRNA ORFs that are not shared by uORFs—most notably, an absence of known association and position with respect to coding regions. The relatively small number of gold standard positive (2-voted) lncRNA ORFs (174) compared with uORFs (741) further complicates development of a predictive score tailored to functional lncRNA ORF prediction.

A large proportion of uORFs in the human genome begin with CTG start codons (19.3%). The greatest number of predicted positive uORFs are also initiated with a CTG start codon (30.0%). ATG has a lower comparative prevalence in the human genome and in the predicted positive set (6.7% and 8.4% respectively) (Figure 3D). Although it is theoretically possible for the uORF and the main ORF (CDS) to share the same stop codon (<10% of uORFs in our genome-wide scan), we excluded N-terminal extensions and annotated alternative translation initiation sites (aTISs) in our analyses as they may retain some function of the primary gene protein product. Thus, all predicted functional uORFs were isolated to the 5′UTR, or overlapping but out of frame with the CDS. The proportion of uORFs ultimately identified as positive from each ribosome profiling study is shown in Figure 3E. The results were similar for each of the ribosome profiling experiments, ∼70% in each case (72% of Gao *et al.*, 71% of Lee *et al.*, 70% of Fritsch *et al.*).

As a validation of our technique, we serially excluded one of three ribosome profiling experiments from the positive training set, instead including the excluded set among unlabeled examples for subsequent retrieval (Figure 3F). The AUC for each of the ROC curves corresponding to these trials is similar: 0.77, 0.82, and 0.79. This shows that we are able to recover experimentally determined uORFs based on our training model. This also suggests a high false-negative rate for ribosome profiling studies as seen by the low overlap observed between ribosome profiling experiments.

**Figure 4.**
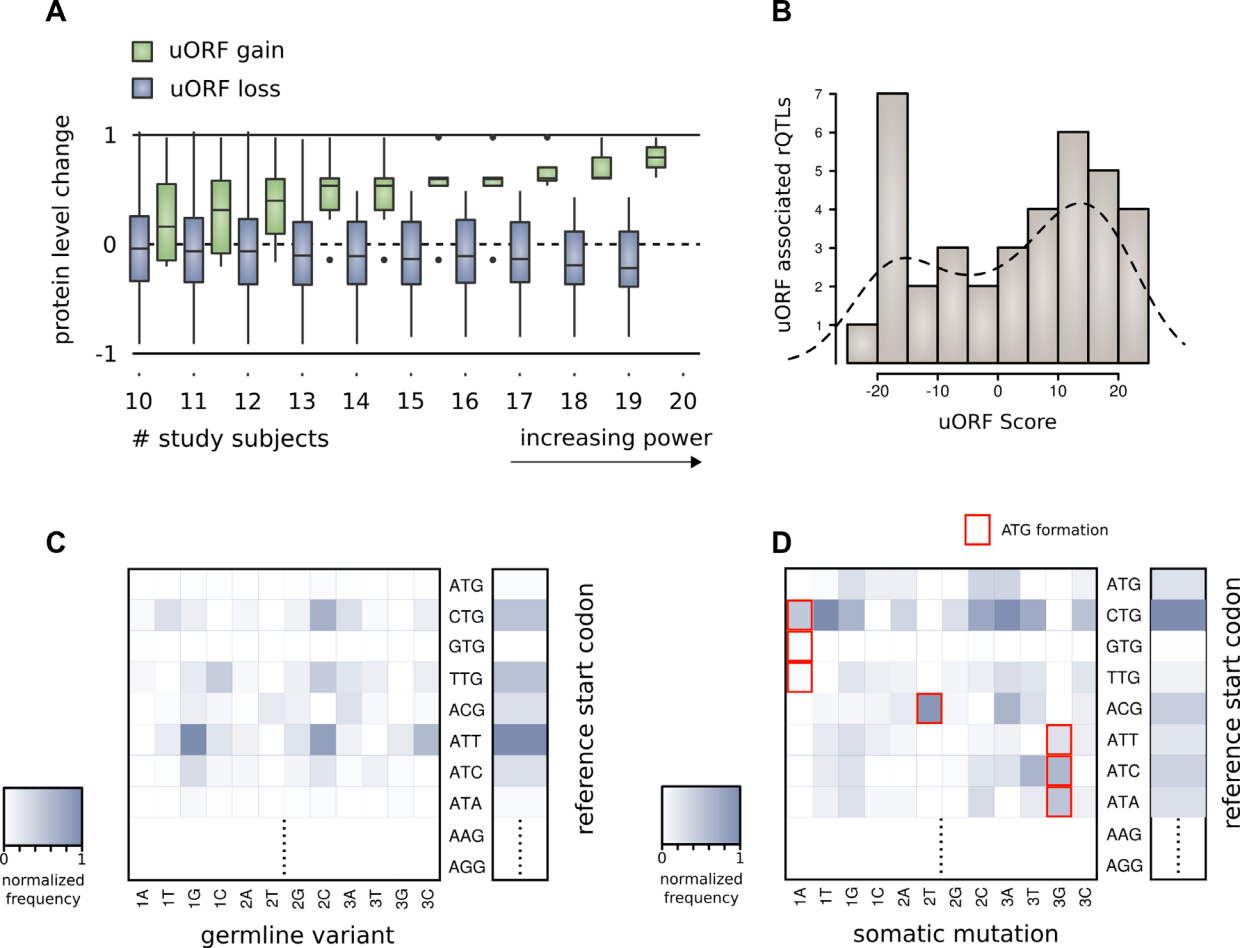
(**A**) Gene level protein expression change for individuals with variants interrupting predicted positive uORFs. The work of Battle *et al.* includes proteomic measurements for 46 individuals with whole genome variant calling through the 1000 Genomes Project. For these individuals, uORF gain is associated with increased protein levels from the downstream gene, while uORF loss is associated with decreased protein levels. (**B**) rQTLs interrupting uORFs according to the score of the corresponding uORF. rQTLs identified by Battle *et al.* display a tendency to hit predicted positive uORFs. (**C**) Density matrix showing the distribution of 1000 Genomes variants that interrupt predicted positive uORF start codons. The vertical axis displays the reference start codon, and the horizontal axis shows the interrupting variant (position—1, 2, 3 – and codon—A, T, G, C). (**D**) Density matrix showing the distribution of somatic mutations found in exomic tumor samples that interrupt predicted positive uORF start codons. The vertical axis displays the reference start codon, the horizontal axis shows the interrupting variant (position—1, 2, 3—and codon—A, T, G, C). ATG forming mutations are highlighted.

As experimental validation of our technique, we examined how natural variation affecting our predicted active uORFs alters protein level and ribosome localization in humans. We hypothesized that an active uORF altered by naturally occurring variants should create observable effect on ribosome occupancy and protein levels from that gene. The results of Battle *et al.*, supplemented by genotype information from the 1000 Genomes Project, provide the basis for validation of our predictions in 46 human subjects (Supplemental Tables S7 and S8). In this study of natural variation amongst humans, SNVs causing gain of predicted positive ATG or CTG uORFs are associated with increase in downstream protein expression. Variants that cause loss of predicted positive uORFs are associated with decrease in downstream protein expression (Figure 4A). That is, there is a statistically significant difference in mean protein expression between variants causing uORF gain compared with uORF loss, among variants with approximate balance between individuals with and without the variant (*N*_loss_ = 133, *N*_gain =_ 17, *t* = 2.6, DOF = 307, *P* = 0.011, for variants shared by >10 individuals). This result is contradictory to the expectation that uORFs generally repress translation of protein downstream. A case study documenting the consequences of uORF gain for the *EIF5A* gene is provided in *Supplemental Results* (Supplementary Figure S1).

We hypothesized that the observation of decreased protein levels upon uORF loss may relate to uORF–uORF repression: an uORF upstream of another uORF may repress the downstream uORF. The loss of one uORF allows a second uORF to act as a translational repressor of the downstream CDS. When we restricted our protein level analysis to uORFs least likely to repress downstream uORFs–uORFs directly overlapping the CDS – we observed a trend towards increases in protein levels with uORF loss (*N*_CDSoverlap_ = 34, μ = 0.065; *N*_CDSnon-overlap_ = 99, μ = –0.055; *P* = 0.097, for variants shared by >10 individuals). This is consistent with the classical role of uORFs as translational repressors.

The above analyses of the effect of uORF alteration on protein level were completed for short insertions and deletions (indels) in addition to SNVs. However, there is an additional challenge to interpreting the effect of indels affecting uORFs – indels have the potential to introduce multiple competing effects (e.g. simultaneous uORF gain and loss) and represent less than 3% of variants in the 1000 Genomes Project variant set examined. These results are provided separately in Supplemental Table S9.

Functional annotation clustering of genes associated with variants affecting predicted positive uORFs showed greatest enrichment for ribosomal proteins including *RPL24* (32 associated with uORF loss and 17 associated with uORF gain) and ribosome associated proteins including *EIF3* (DAVID enrichment score 20.94, *N*_terms_ = 12, *N*_genes(enrich.)_/*N*_genes(tot.)_ = 108/961, *p*_mean(geom.)_ << 0.001). *EIF3* and ribosomal proteins like *RPL24* are thought to overcome uORF mediated repression in *Arabidopsis thaliana* through facilitation of translation reinitiation (70).

For these same 46 human subjects, cis-rQTLs provide an inventory of variants with statistically significant effect on local ribosome occupancy. There is significant enrichment for rQTLs interrupting positively scored start codons (Figure 4B). If mutations hit uORFs randomly, 14.9% of the time they would hit a positively scored uORF. However, we observe that 48% of these rQTLs (21/44) interrupt positively scored start codons—a 3× higher rate. This indicates that many rQTLs may measure the direct effect of disruption of functional uORFs.

The ATG start codon is relatively conserved among predicted positive start codons—it is rarely interrupted by 1000 Genomes Project variants (relative rate (RR) 0.03), suggesting its functional importance. This is consistent with prior analyses, which find that ATG is the most conserved upstream start codon (71). The CTG start codon, although more prevalent among predicted positive uORFs, is altered relatively frequently by natural human variants (RR 0.52) (Figure 4C). In exomic tumor samples from cancer patients, CTG is the most commonly modified predicted positive uORF start codon. ATG is interrupted at a RR of 0.25 in comparison to CTG (Figure 4d). The higher RR of interruption of both ATG and CTG in cancer as compared to germline variants—8-fold higher, and 2-fold higher respectively—further suggests functional consequences attributable to these uORFs. Ultra-rare (singleton) variants from the ExAC catalog v.1 were also scanned for alteration of uORF predicted positive start codons. The singleton variants from the ExAC catalog affecting predicted positive uORFs help highlight high-impact germline mutations affecting conserved uORF start codon sites (Supplemental Table S10).

Exomic cancer mutations breaking the highest scored uORFs are listed in Supplemental Table S11. These mutations interrupt uORFs associated with well-studied oncogenes and tumor suppressors. *MYC* and *BCL2* are the two genes associated with the greatest recurrence of uORF interruptions, and we identify recurrent mutation of positively scored uORFs associated with *PTEN, TP53, ERCC1* and *MSH5*. An annotation of Supplemental Table S11 is provided indicating somatic variants that affect uORFs associated with COSMIC cancer genes, as well as recurrent somatic variants affecting uORFs that are recurrent across patient samples. These annotations indicate variants that are priorities for future follow-up analyses. We found that predicted functional uORFs located on genes associated with cell survival and cell differentiation are among those most frequently disrupted by mutation in cancer compared to expectation, suggesting an impact on tumor cell fitness (Supplementary Figure S2). Rates of predicted functional uORF interruption varied significantly across cancer types, indicating that uORF disruption has greater functional impact in certain cancers (Supplementary Figure S3). Genome-wide association study (GWAS) SNVs listed in the NHGRI-EBI GWAS database that impact our predicted uORFs are listed in Supplemental Table S12. GWAS diseases associated with SNVs interrupting positively scored uORFs include prevalent chronic conditions like asthma (rs3771180), and type 2 diabetes (rs1552224). We further scanned variants from the Human Gene Mutation Database (HGMD) of published human inherited disease mutations (Supplemental Table S13) and the ClinVar database of variants with human phenotypic correlations (Supplemental Table S14). These variant analyses implicate the possible influence of uORF alteration in a number of additional disease conditions such as Parkinson's Disease and cancer-predisposing Lynch Syndrome.

Additional variants associated with susceptibility and prognosis in cancer are found to interrupt positively scored uORFs, like rs779805 upstream of the *VHL* gene, and rs34330 upstream of *CDKN1B*. Although linkage disequilibrium and overlap among regulatory elements complicates interpretation of these GWAS studies, these disease-associated SNVs may owe their functional consequence to alteration of a translated uORF.

## DISCUSSION

In this study, we identify 188,802 likely active upstream open reading frames from a genome-wide set of 1,270,265 unique uORFs. We further highlight the 10% of our predictions that are most likely to be functional as a high reliability subset.

We began by assuming that ribosome profiling experiments have a high false negative rate for identification of functional uORFs. Our method applied the intersection of three ribosome profiling studies to form a reference set of known active uORFs. The low overlap between ribosome profiling experiments suggests a high false-negative rate in individual experiments. The finding that pairs of ribosome profiling experiments may be used to correctly identify the uORFs translated in a third experiment also suggests a high false negative rate. The large number of uORFs we identified as likely functional is consistent with this premise, but significant in comparison to other studies on the topic.

There is precedent for our findings in comparisons of large-scale parallel experiments of interaction between biomolecules. The protein-protein interaction experiments of Uetz *et al.* aimed to produce a comprehensive, genome-wide map of protein interactions (72). Subsequent experiments by Ito *et al.* with similar technique and scope showed low overlap with the results of Uetz *et al.* (73). It became clear that the universe of possible protein-protein interactions is much larger than identified in either experiment individually. Combining datasets improves the identification of these protein interactions (74).

Our use of an intersection between ribosome profiling experiments provides some control against differences in experimental conditions and tissue specific results (three experiments and both HEK293 and THP-1 cells were examined). However, just as protein levels vary widely across cell-types (75), it may prove that the activity of uORFs varies considerably across cell types and cellular conditions. Analysis of cell-type specific and condition specific activity of uORFs may further expand estimates of the population of translated uORFs.

Our study helps clarify how attributes of structure and context of a given uORF—including start codon, base composition, and relative position to the CDS—likely contribute to varying functionality among uORFs. Although ATG is the most common uORF start codon identified in ribosome profiling experiments, lower affinity near cognate-start codons may have great functional impact on the landscape of translation due to their overall abundance.

An important validation of our predictions is the finding that alteration of predicted functional uORFs, as a consequence of germline genetic variation, impacts ribosome binding and protein levels in humans. Generally, we assume that uORFs act as translational repressors. However, the overall effect of uORF loss appears to be a decrease in downstream protein level. This is contrary to common view that uORFs act as translational repressors. Mechanisms have been studied where uORFs act to up-regulate expression of a downstream coding sequence (e.g. leaky-scanning, and translation reinitiation). Ribosomal reinitiation at an uORF on the ATF4 gene is one particularly well studied example of such a mechanism (76). Our analysis suggests that positive effect on translation may be a more common consequence for upstream open reading frames than was previously credited.

The protein level changes we observed may also relate to multiple indirect effects of uORF repression such as (i) uORF–uORF interaction where one uORF acts to repress another uORF, (ii) variation affecting overlapping uORFs simultaneously and (iii) uORFs upstream of coding genes that themselves regulate translation. Indeed, the observation of enrichment of translational mediators and ribosomal proteins among our uORFs affected by genetic variation, suggests the possibility of cascading functional effects related to uORF gain or loss. Furthermore, among genes with multiple predicted positive uORFs, the presence of CDS-overlapping uORFs resulted in opposite effect on CDS translation compared to those uORFs entirely upstream of the CDSs. This observation suggests that the effect of interaction among uORFs is worthy of further study.

In addition to our examination of uORFs on protein coding genes, we also observed significant translation initiation on lncRNA ORFs replicated across the studies we examined. As has been noted by other investigators, these ORFs may serve a function similar to uORFs—regulating translation from as yet undiscovered downstream coding sequences on lncRNAs. Alternatively, these lncRNA ORFs may themselves function as CDS regions, encoding short novel peptides that have been mis-annotated as non-coding (77). Our work supports a need for further investigation of the coding potential of these ‘non-coding’ transcripts.

Limitations to our catalog include the possibility that post-transcriptional RNA editing could result in additional uORFs. Also, the mechanism of repeat associated non-ATG translation, whereby translation may initiate at RNA repeat motifs in absence of a specific start codon, suggests there may exist uORFs that do not initiate translation at one of the near-cognate start codons we examined (78). Future work may be aimed towards clarifying to what extent these classes of translated uORFs exist.

Our results suggest avenues for future research. Identification of human germline variants altering predicted positive uORFs reveals locations where the creation or destruction of an uORF is likely to alter protein levels. Employing this method, we identified disease-associated SNVs, including a number of GWAS SNVs, that likely owe their significance to alteration of a functional uORF. Our work could be used to help broaden knowledge of the role of uORFs in cancer beyond recently identified individual examples (79). Finally, we provide a catalog that can serve as a point of reference for other researchers engaged in the investigation of uORF function.

## DATA AVAILABILITY

Source code and resources related to this analysis are made publicly available in a GitHub repository: https://github.com/gersteinlab/uORFs.

## Supplementary Material

Supplementary Data
