# Supplementary material for: A comprehensive catalog of predicted functional upstream open reading frames in humans
Source: Nucleic Acids Res. 2018 Mar 19;46(7):3326–38. doi: 10.1093/nar/gky188 (PMC6283423; doi:10.1093/nar/gky188)
Supplement: Supplementary Data [file gky188_supplemental_files.zip › Supplement_uORFs.docx]

**SUPPLEMENT CONTENTS:**

1. **SUPPLEMENTAL RESULTS:**
   1. **Case study of uORF gain associated with gene EIF5A [Fig. S1].**
   2. **GO term annotation of uORFs interrupted in cancer [Fig. S2].**
   3. **Analysis of uORF mutation across cancer types [Fig. S3].**
2. **SUPPLEMENTAL DATA FILES:**

**2.1. Supplemental Table S1: uORF feature descriptions**

**2.2. Supplemental Table S2: uORF feature ranking via Kolmogorov–Smirnov statistic.**

**2.3. Supplemental Table S3: uORFs with peptide mappings.**

- 1. **Supplemental Table S4: Predicted translated uORFs.**
  2. **Supplemental Table S5: Predicted translated uORFs (highest scoring 10%).**
  3. **Supplemental Table S6: Translated lncRNA ORFs**
  4. **Supplemental Table S7: uORF loss effect on protein level.**
  5. **Supplemental Table S8: uORF gain effect on protein level.**
  6. **Supplemental Table S9: uORF indel effect on protein level.**
  7. **Supplemental Table S10: ExAC singleton variants affecting predicted positive uORFs.**
  8. **Supplemental Table S11: Somatic mutation affecting predicted positive uORFs.**
  9. **Supplemental Table S12: GWAS variants affecting predicted positive uORFs.**
  10. **Supplemental Table S13: HGMD variants affecting predicted positive uORFs.**
  11. **Supplemental Table S14: ClinVar variants affecting predicted positive uORFs.**

**1. SUPPLEMENTAL RESULTS:**

**2.1.** *Case study of uORF gain associated with gene EIF5A:*


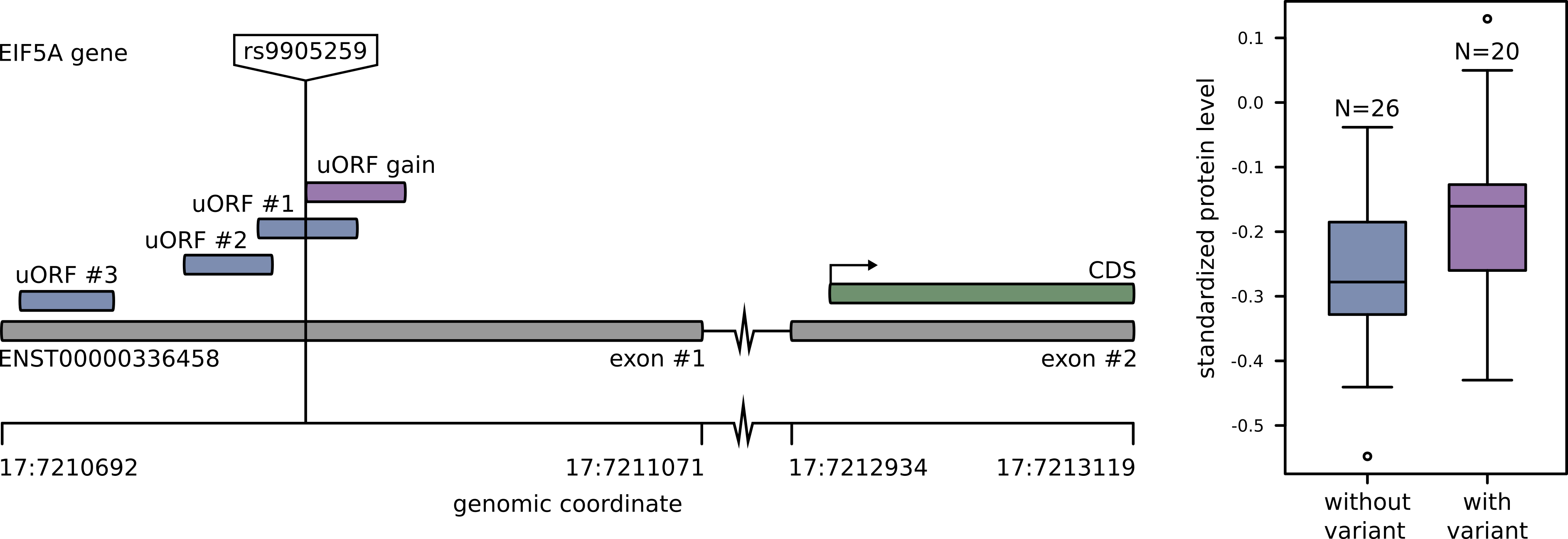


***Figure S.1. uORF gain associated with increased gene expression for EIF5A and variant rs9905259.*** *Predicted positive ATG uORFs associated with the reference genome are numbered 1-3 (Ensembl transcript ENST00000336458, EIF5A-001). A gained predicted positive ATG uORF associated with rs9905259 is positioned downstream of these three other ATG uORFs. Variant rs9905259 also intersects another uORF on the same transcript, altering its amino-acid sequence. An increase in EIF5A protein expression is observed for individuals with this variant, compared to individuals without the variant.*

EIF5A has 3 predicted positive upstream open reading frames (uORFs) with ATG start codons (GRCh37; EIF5A-001). Variant rs9905259, with minor allele frequency 0.05, introduces an additional predicted positive ATG uORF downstream of all other ATG uORFs [Figure S.1]. The overall effect of this additional uORF is an increase in downstream EIF5A protein level (N_reference_=25, μ_expression_=-0.241764318; N_rs9905259_=21, μ_expression_=-0.17162073; p= 0.044)

It is possible that uORFs upstream of this gained uORF repress this uORF. Furthermore, in addition to introducing an uORF, variant rs9905259 intersects an existing uORF. Thus, with the introduction of an uORF, there is simultaneous alteration of the amino-acid sequence of another uORF. For this reason, there may by multiple uORF related functional consequences resulting from rs9905259. Finally, EIF5A encodes a protein that is involved in translation elongation, and possibly mRNA degradation(1, 2). There is possibility of a regulatory cascade, where uORF mediated regulation of translation affects a gene that itself regulates translation.

**2.2.** *Gene Ontology (GO) enrichment of uORFs interrupted in cancer:*

*
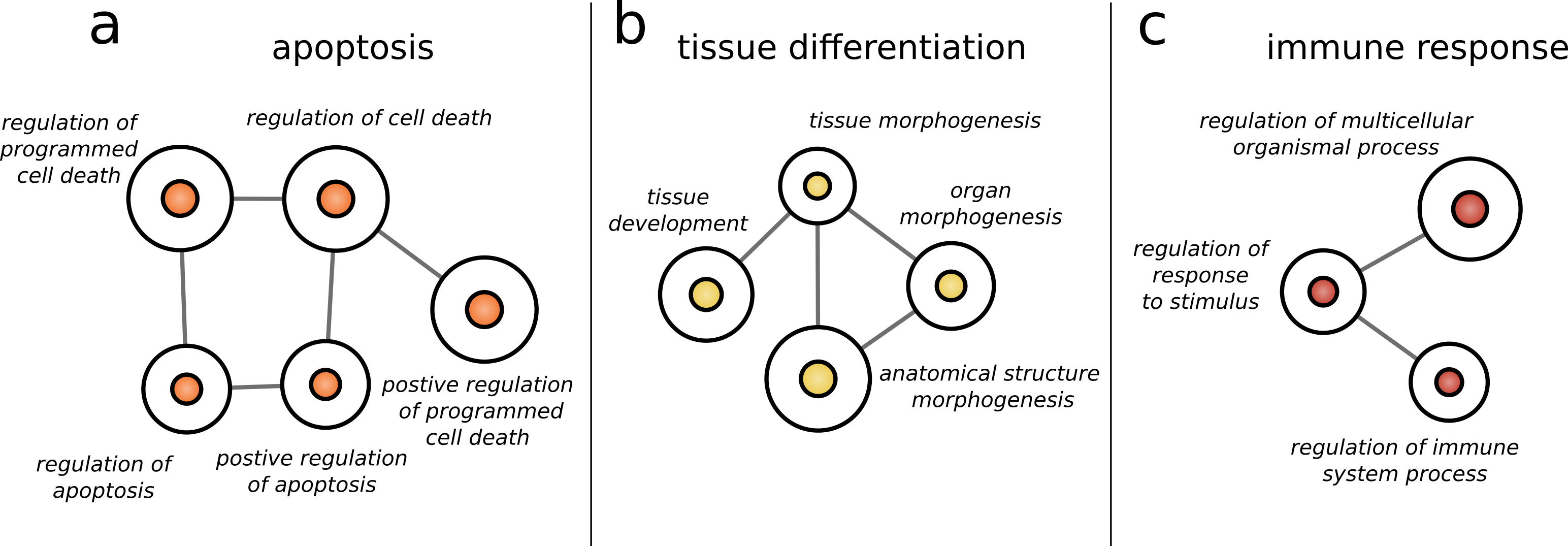
*

***Figure S.2. GO/PANTHER terms, for statistically overrepresented genes with uORF start codons interrupted by somatic variants in tumor samples (Alexandrov et al.).*** The size of each node corresponds to the number of uORFs associated that GO term. Thresholds were established to eliminate relatively common GO terms (>1250 associated uORFs), and relatively uncommon GO terms (<250 associated uORFs). 3 principle networks emerge: a) tissue morphogenesis, b) immune function, and c) apoptosis. Networks were developed using the statistical package BiNGO and include adjustment for multiple hypothesis testing.

Patterns of function in genes affected by mutation of uORFs in cancer was assessed via the GO genome annotation database (3). Overrepresented GO terms were identified, with overrepresentation assessed via the hypergeometric statistical test with multiple testing correction via Benjamini & Hochberg's False Detection Rate (FDR) correction (4). Networks between GO terms were constructed using the Cytoscape package BiNGO (5) [Figure S.2].

Three networks of overrepresented GO terms remain following correction for statistical significance and multiple testing. These are networks associated with cellular functions of probable significance in cancer -- cellular death, immune modulation, and tissue morphogenesis. Lack of response to apoptotic signaling, and immune tolerance, are well known mechanisms by which cancer cells prolong survival. The alteration of genes involved in tissue morphogenesis, may relate to the poor tissue differentiation exhibited by cancer cells.

**2.3.** *Analysis of uORF mutation across cancer types:*


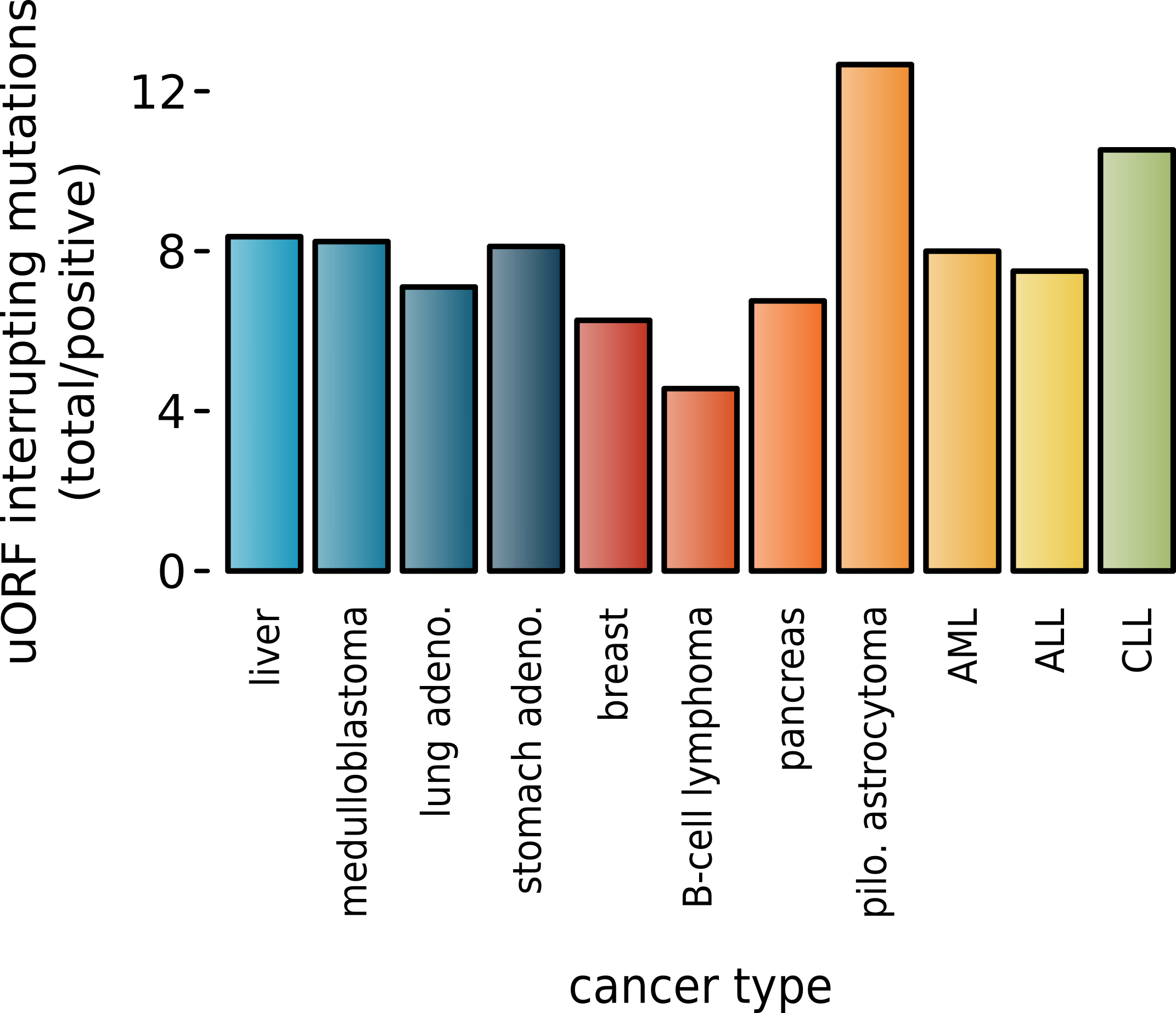


***Figure S.3. uORF score ratio (# total / # positive) for uORFs interrupted by mutation in cancer according to cancer type.***

In order to evaluate the frequency with which uORFs are interrupted by mutation in cancer, the proportion of positive uORFs interrupted by mutation was calculated for each cancer type [Figure S.3]. The proportion of positively scored uORFs to negative scored uORFs varied across cancer types, ranging from a low of 1:12.7 for pilocytic astrocytoma, to a high of 1:4.6 for B-cell lymphoma (chi-square = 45, p-value = <0.001). This finding suggests that B-cell lymphoma or breast cancer, may depend to a greater extent on altered uORFs for increased cellular fitness, than pilocytic astrocytoma or CLL.

**REFERENCES:**

1. Saini,P., Eyler,D.E., Green,R. and Dever,T.E. (2009) Hypusine-containing protein eIF5A promotes translation elongation. *Nature*, **459**, 118–121.

2. Park,M.H., Nishimura,K., Zanelli,C.F. and Valentini,S.R. (2010) Functional significance of eIF5A and its hypusine modification in eukaryotes. *Amino Acids*, **38**, 491–500.

3. Gene Ontology Consortium,T., Ashburner,M., Ball,C.A., Blake,J.A., Botstein,D., Butler,H., Michael Cherry,J., Davis,A.P., Dolinski,K., Dwight,S.S., *et al.* (2000) Gene Ontology: tool for the unification of biology. *Nat. Genet.*, **25**, 25–29.

4. Author,T., Benjamini,Y., Hochberg,Y. and Benjaminit,Y. (1995) Controlling the False Discovery Rate: A Practical and Powerful Approach to Multiple Controlling the False Discovery Rate: a Practical and Powerful Approach to Multiple Testing. *J. R. Stat. Soc. Ser. B*, **57**, 289–300.

5. Maere,S., Heymans,K. and Kuiper,M. (2005) BiNGO: a Cytoscape plugin to assess overrepresentation of Gene Ontology categories in Biological Networks. *Bioinformatics*, **21**, 3448–3449.
